# Efficacy and safety of radiation therapy in advanced adrenocortical carcinoma

**DOI:** 10.1038/s41416-022-02082-0

**Published:** 2022-12-08

**Authors:** Otilia Kimpel, Paul Schindler, Laura Schmidt-Pennington, Barbara Altieri, Felix Megerle, Harm Haak, James Pittaway, Ulrich Dischinger, Marcus Quinkler, Knut Mai, Matthias Kroiss, Bülent Polat, Martin Fassnacht

**Affiliations:** 1 Division of Endocrinology and Diabetes, Department of Medicine, University Hospital, University of Würzburg, Würzburg, Germany; 2 Department of Endocrinology & Metabolism, Charité—Universitätsmedizin Berlin, corporate member of Freie Universität Berlin, Humboldt-Universität zu Berlin, and Berlin Institute of Health, 10117 Berlin, Germany; 3 Department of Internal Medicine, Máxima MC, Eindhoven/Veldhoven The Netherlands. Maastricht University, CAPHRI School for Public Health and Primary Care, Ageing and Long-Term Care, Maastricht, the Netherlands. Department of Internal Medicine, Division of General Internal Medicine, Maastricht University Medical Centre+, Maastricht, the Netherlands; 4 Centre for Endocrinology, William Harvey Research Institute, Barts and the London School of Medicine and Dentistry, Queen Mary University of London, London, UK; 5 Endocrinology in Charlottenburg, Berlin, Germany; 6 Department of Medicine IV, University Hospital, LMU Munich, Ziemssenstraße 1, 80336 München, Germany; 7 Department of Radiation Oncology, University Hospital, University of Würzburg, Würzburg, Germany; 8 Comprehensive Cancer Center Mainfranken, University of Würzburg, Würzburg, Germany

**Keywords:** Adrenal tumours, Adrenal tumours

## Abstract

**Background:**

International guidelines emphasise the role of radiotherapy (RT) for the management of advanced adrenocortical carcinoma (ACC). However, the evidence for this recommendation is very low.

**Methods:**

We retrospectively analysed all patients who received RT for advanced ACC in five European centres since 2000. Primary endpoint: time to progression of the treated lesion (tTTP). Secondary endpoints: best objective response, progression-free survival (PFS), overall survival (OS), adverse events, and the establishment of predictive factors by Cox analyses.

**Results:**

In total, 132 tumoural lesions of 80 patients were treated with conventional RT (cRT) of 50–60 Gy (*n* = 20) or 20–49 Gy (*n* = 69), stereotactic body RT of 35–50 Gy (SBRT) (*n* = 36), or brachytherapy of 12–25 Gy (BT) (*n* = 7). Best objective lesional response was complete (*n* = 6), partial (*n* = 52), stable disease (*n* = 60), progressive disease (*n* = 14). Median tTTP was 7.6 months (1.0–148.6). In comparison to cRT_20-49Gy_, tTTP was significantly longer for cRT_50-60Gy_ (multivariate adjusted HR 0.10; 95% CI 0.03–0.33; *p* < 0.001) and SBRT (HR 0.31; 95% CI 0.12–0.80; *p* = 0.016), but not for BT (HR 0.66; 95% CI 0.22–1.99; *p* = 0.46). Toxicity was generally mild and moderate with three grade 3 events. No convincing predictive factors could be established.

**Conclusions:**

This largest published study on RT in advanced ACC provides clear evidence that RT is effective in ACC.

## Introduction

Adrenocortical carcinoma (ACC) is a rare malignancy with poor prognosis and 5-year overall survival ranging from about 80 % in patients with ACC stage I to less than 20 % in stage IV disease [1–8]. However, also in stage IV some patients survive many years and even complete responses have been reported [9–11].

For the treatment of recurrent and metastatic disease not amenable to complete resection, most international reviews and official guidelines recommend, mitotane alone or in combination with chemotherapy as first-line therapy [1, 8, 12–15]. Surgery of metastases is usually only performed if all tumoural lesions can be removed and the interval to previous surgery was above 12 months [16]. However, both current international guidelines on ACC emphasise the role of local therapies in advanced disease [1, 2]. The panellists agreed that local therapeutic measures (including radiation therapy (RT)) are of value for therapy of advanced ACC and suggested an individualised decision on which method to choose based on the localisation of the tumour lesion(s), local expertise, prognostic factors and patient’s preference. RT is also indicated for pain, prevention of imminent metastatic complications, severe mass effect or neurological symptoms. Several small studies and reviews suggest an improvement of neurological symptoms and pain relief by RT in ACC [2, 17–20] similar to other solid tumours [21, 22].

However, if studies focusing only on pain relief are excluded, there are only four retrospective reports including fewer than 60 patients in which 73 treatments with RT are described in unresectable ACC, but only 64 of them could be evaluated [19, 23–25].

Ho et al. investigated RT in a small cohort of 12 patients with advanced ACC treated with 18 courses of RT, but only 13 had adequate follow-up imaging to assess a radiographic response. After these 13 courses, 4 lesions decreased in size by 30% or greater, 2 were stable for the documented follow-up of 9.9 and 2.2 months, whereas 7 lesions were initially stable, but progressed after a mean time of 4.8 months [19]. In a study at MD Anderson in Houston, 19 patients were treated with RT due to abdominal disease or local recurrence. RT was described as only moderately effective. Only 3 patients (15.8%) were classified as responders, which was defined as a significant decrease of the lesion or as stabilisation of the lesion for at least 2 years. Combination treatment with mitotane and RT was moderately effective in three of 10 patients (30%) [23]. Magee et al. investigated the efficacy of RT in patients with locally invasive tumour, metastases or recurrent disease after surgery. Treatment response could be evaluated in only six of 10 patients treated by RT. Four experienced partial response assessed by reduction of the size of the primary tumour or metastases, or biochemical response, whereas in two patients no benefit could be demonstrated. However, the documented responses also lasted fewer than 12 months before disease progression [24]. Sabolch et al. evaluated the impact of RT on local tumour control in unresectable disease and local progression occurred only in 1 of 16 cases [25]. In conclusion, the results in these 57 patients were quite heterogeneous, but one has to acknowledge that the majority of these patients have been treated before 2000 and the used techniques are not really comparable anymore with methods offered to patients nowadays. Furthermore, none of these studies could provide any data on prognostic or predictive factors.

There is more experience for treatment with RT in an adjuvant setting. Until now retrospective studies describing more than 500 cases have been reported [12, 24–32], but several publications used the same databases with significantly overlapping cohorts and the true number of patients in large series is probably only around 380. Almost all studies showed that adjuvant RT can reduce the risk of local recurrence. However, data are conflicting regarding overall recurrence-free and overall survival [33]. Nevertheless, these studies suggest that the majority of ACCs are sensitive to RT.

Therefore, the aim of this study was to investigate patients with advanced ACC treated with currently state of the art RTs and to evaluate its efficacy and tolerance, and to identify predictors of response to RT.

## Subjects and methods

### Study population

This cohort study was part of the ENSAT registry study (www.ensat.org/registry) in five European reference centres for ACC (Berlin, Germany; Munich, Germany; Würzburg, Germany; London, UK and Eindhoven, Netherlands). It was approved by the ethics committees or institutional review boards at all participating institutions and all patients provided written informed consent. Patients with advanced ACC (defined as not completely resectable disease) and treatment with RT were included if they were treated between 2000 and April 2020. Follow-up for this study was closed in July 2022.

Demographic, clinical, and histological parameters sex, age at diagnosis, tumour size, evidence of hormonal excess, ENSAT tumour stage [7], information on local and systemic therapies before RT and details on RT (see below) were retrieved from the ENSAT ACC registry and medical records. All histological diagnoses were confirmed by experienced pathologists. Tumour staging at diagnosis was based on imaging studies and by the findings during surgery and pathological examination. Autonomous cortisol excess was defined as pathological dexamethasone test in the presence of suppressed baseline plasma ACTH.

Patients with lack of relevant information on primary diagnosis or follow-up or newly started concomitant systemic anti-tumour treatment within 12 weeks prior RT (except progressive disease was already documented) were excluded. Ongoing mitotane treatment (started more than 12 weeks prior RT) was permitted and mitotane blood levels were documented. Patients treated with adjuvant RT did not qualify for this study.

### Details on radiotherapy

The following information on RT was captured: RT modality classified as conventional RT (cRT) stereotactic body radiotherapy (SBRT), and brachytherapy (BT), first day and duration of RT, number of fractions, and dose per fraction in Gray (Gy). Due to different RT techniques and treatment doses, patients were divided in different groups for statistical analyses. We defined four groups according to modality and dosage: patients treated by cRT with 50–60 Gy (cRT_50-60Gy_) or 20–49 Gy (cRT_20-49Gy_), by SBRT (with 35–50 Gy) and by BT (with 12–25 Gy). In addition, we analysed patients according to the equivalent dose in 2 Gy fractions (EQD2) and the biologically effective dose (BED10) using the linear quadratic model for radiobiology with an alpha/beta ratio of 10 Gy for adrenocortical tumours. For EQD2 and BED10 the patients were distributed to three groups according to RT dose. Since this retrospective analysis did not allow the definition in all patients whether a definitive or a palliative approach was intended at the time of RT, we discriminated post hoc two different groups: all procedures applying cRT_50-60Gy_, SBRT or BT were classified as ‘potentially definitive approach’, whereas cRT_20-49Gy_ was judged as ‘potentially palliative approach’.

### Outcome assessment

Prior to any analysis, we defined time to progression of the treated lesion (‘tTTP’) as the most relevant outcome. Each lesion was evaluated separately. We further analysed overall progression-free survival (‘oPFS’) which means that all tumoural lesions were judged independent of treatment with RT. The judgement of treatment response was based on routine radiologic assessment applying RECIST 1.1 criteria as close as possible. Best objective response was defined according to RECIST 1.1 criteria. The first radiological evaluation was performed after 2.6 months (range 1.1–14.6) and the second after 5.6 (1.5–17.8) months.

### Documentation of adverse events

Medical records were reviewed for adverse events associated with RT. All adverse events were retrospectively scored according to the toxicity criteria of the radiation therapy oncology group (RTOG) and the common terminology criteria for adverse events (CTCAE v5.0) [34].

### Statistical analysis

Time to progression of the treated lesion (tTTP) was defined as the time elapsing from the first day of RT to the first evidence of progression of this lesion or the date of last follow-up. Overall progression-free survival (oPFS) was defined as the time between the start of RT and the date of progression of any lesion or last follow-up. Overall survival (OS) was defined as the time from the date of first RT to the date of death or last follow-up. Patients without progression or death were censored at the date of last follow up. Survival analysis was calculated using the Kaplan-Meier method, and differences between groups were assessed by log-rank statistics.

We performed univariable analysis of factors that could potentially influence outcome after RT: sex, age, RT modality (cRT_20-49Gy_, cRT_50-60Gy_, SBRT, BT), time interval between primary diagnosis and RT (≤12 months vs. >12 months), number of therapies (in addition to primary surgery) before RT (≤3 vs. >3), size of the tumoural lesion treated with RT (≤30 mm vs. >30 mm), Ki67 index of the primary tumour (≤15 % vs. >15%), presence of autonomous glucocorticoid excess (yes vs no), localisation of treated lesion, number of lesions ≤5 vs. <5 and concomitant mitotane treatment (maximum plasma level during RT ≤ 14 mg/l vs. >14 mg/l). In a multivariable approach using the Cox proportional hazards model, tTTP, oPFS and OS were adjusted for all factors with *p* < 0.1 in univariate analysis. As an alternative to RT modality, EQD2 and BED10 were analysed in the same manner.

All reported *P* values are two-sided and *P* < 0.05 were considered to indicate statistical significance. Data were analysed using SPSS v.26 (IBM SPSS Statistics).

## Results

### Patient characteristics

The total cohort consisted of 80 patients with 132 individual lesions treated with RT. Key patients’ characteristics are given in Table 1. All patients suffered from advanced ACC at the time they were treated with RT. Age, sex, glucocorticoid excess, ENSAT stage and resection status at primary diagnosis did not differ significantly between the four groups of RT modality, but median Ki67 index of the primary tumour was higher in the cRT_20-49_ and ‘brachytherapy’ group compared to the others.

**Table 1:** Baseline characteristics of the patients.

	cRT_20-49 Gy_ *n* = 44	cRT_50-60 Gy_ *n* = 16	SBRT *n* = 16	BT *n* = 4	*P*
Median age - years (range)	49.5 (18–74)	49.5 (18–78)	46.0 (26–66)	42.0 (35–53)	0.52
Sex - n (%)					
Male	15 (34.1)	6 (37.5)	5 (31.3)	1 (25.0)	0.96
Female	29 (65.9)	10 (62.5)	11 (68.7)	3 (75.0)	
Glucocorticoid excess - n (%)	11 (25.0)	4 (25.0)	4 (25.0)	1 (25.0)	1.0
ENSAT stage at primary diagnosis - n (%)					0.81
1	3 (6.8)	1 (6.3)	2 (12.5)	0.0	
2	18 (40.9)	9 (56.3)	8 (50.0)	1 (25.0)	
3	12 (27.3)	3 (18.8)	2 (12.5)	2 (50.0)	
4	11 (25.0)	2 (12.5)	3 (18.8)	1 (25.0)	
Resection status at primary diagnosis - n (%)					0.45
0	24 (54.5)	14 (87.5)	9 (56.3)	2 (50.0)	
1	3 (6.8)	1 (6.3)	2 (12.5)	0.0	
2	5 (11.4)	0.0	1 (6.3)	0.0	
X	8 (18.2)	1 (6.3)	4 (25.0)	2 (50.0)	
Median Ki67 index of the primary tumour -% (range)	20.0 (5–60)	10.0 (2–80)	10.0 (3–60)	20.0 (5–70)	0.018

In total 80 patients, 6 patients are represented twice due to therapy with two different RT modalities (cRT = conventional RT, SBRT = stereotactic body radiation therapy, BT = brachytherapy).

### Radiotherapy characteristics

Table 2 provides details of the RT modalities and the treated lesions. Of a total of 132 tumoural lesions, 69 were treated with cRT_20-49Gy_, 20 with cRT_50-60Gy_, 36 with SBRT and 7 lesions with BT. Due to the different RT modalities the median number of fractions, the median dose per fraction, the median EQD2 and median BED10 differed significantly between the groups. Furthermore, lesions treated with SBRT were the smallest and lesions treated with brachytherapy were the largest. Median time between start of RT and first or second imaging was not significantly different between groups. Furthermore, the time interval between RT and first and second tumour evaluation were similar between both groups.

**Table 2:** Baseline characteristics of RT.

	cRT_20-49 Gy_ *n* = 69	cRT_50-60 Gy_ *n* = 20	SBRT *n* = 36	BT *n* = 7	*P*
Localization of radiated lesion - n					
Local recurrence	6	10	6	0	
Lung	7	4	21	0	
Liver	6	1	2	7	
Bone	44	2	0	0	
Lymph node	1	1	1	0	
Brain	1	0	3	0	
Other soft tissue	4	2	3	0	
Median size of lesion - mm (range)	39.5 (7–190)	29.0 (12–135)	20.0 (7–52)	55.0 (24–140)	<0.001
Median dose per fraction – Gy (range)	3.0 (1.2–8)	2.1 (1.8–4)	12.5 (4–26)	15.0 (15–20)	<0.001
Median number of fractions – n (range)	12.0 (1–28)	27.0 (15–30)	3.5 (1–10)	1.0 (1–1)	<0.001
Median EQD2 – Gy (range)	36.5 (12–49.6)	54.6 (49.6–70.0)	70.3 (43.8–134.8)	31.3 (31.3–50.0)	<0.001
Median BED10 – Gy (range)	43.8 (14.4–59.5)	65.5 (59.5–84.0)	84.4 (52.5–161.7)	37.5 (37.5–60.0)	<0.001
Median number of therapies (in addition to primary surgery) before RT – n (range)	2.0 (0–17)	3.0 (1–5)	4.0 (1–17)	7.0 (1–10)	<0.001
Median time between primary diagnosis and start of RT – months (range)	23.8 (0.6–151.9)	39.7 (1.1–158.1)	43.9 (3.7–260.7)	28.7 (17.7–67.1)	0.016
Median number of lesions not treated with RT- n (range)	4.0 (0–16)	1.5 (0–9)	5.0 (0–9)	3.0 (0–8)	0.34
Concomitant mitotane during RT – n (%)	45 (65.2)	11 (55.0)	23 (63.9)	4 (57.1)	0.85
Mitotane plasma level >14 mg/L during RT					
Yes (%)	21 (30.4)	3 (15)	11 (30.6)	3 (42.9)	0.32
No (%)	24 (34.8)	5 (25)	12 (33.3)	1 (14.3)	
Median time to first imaging – months (range)	2.7 (1.0–14.7)	2.7 (1.9–6.9)	2.1 (1.4–5.2)	2.5 (0.6–3.7)	0.42
Median time to second imaging –months (range)	5.8 (1.5–17.9)	5.7 (3.6–13.8)	5.4 (2.5–8.6)	3.5 (1.2–7.5)	0.12

In total 132 tumoural lesions were treated with RT (cRT = conventional RT, SBRT = stereotactic body radiation therapy, BT = brachytherapy).

### Clinical outcome according to treatment groups

57 of 80 patients died during follow-up (all due to progressive disease). Median time of follow-up of alive patients was 21.5 (1.9–132.2) months.

**Fig. 1:**
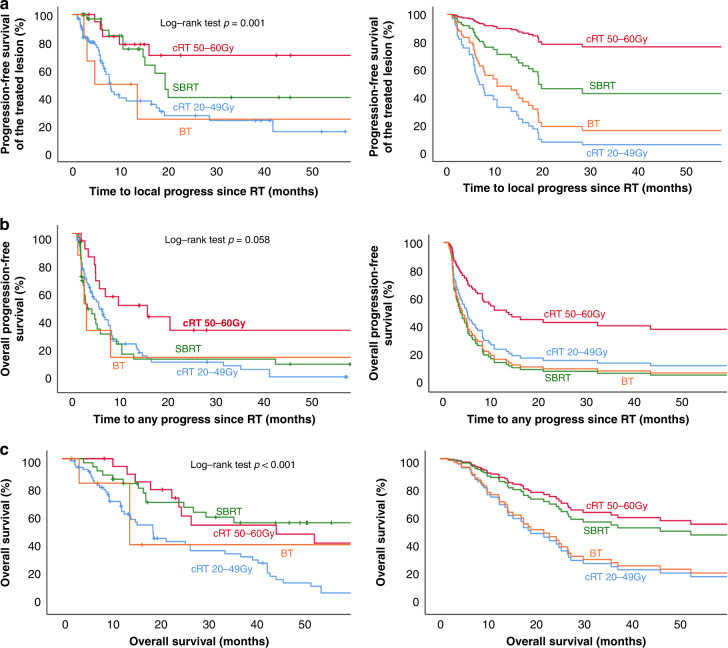
Time to local progress, time to any progress and overall survival since RT in all patients with advanced ACC treated with RT. Kaplan-Meier (right panel) and Cox regression (left panel) survival curves (**A**) for time to progress of the treated lesion (tTTP), (**B**) overall progression free survival (oPFS) and (**C**) overall survival (OS).

Best objective response of the 132 irradiated lesions was complete response in 6 lesions (4.5%), partial response in 52 lesions (39.4%) and stable disease in 60 (45.5%). Progression in the subsequent imaging was present in only 14 lesions (10.6%). The objective responses were scattered among the different treatment groups (Table 3). However, tTTP was significantly different between groups (Fig. 1). For the cRT_50-60_ Gy median tTTP was not reached, whereas it was 19.3 months for the SBRT group, 7.9 months for the cRT_20-49_ Gy group and only 4.6 months in patients treated with BT (p < 0.001).

**Table 3:** Objective response according the different treatment groups.

	Number of lesions *n*	Complete response *n* (%)	Partial response *n* (%)	Stable disease *n* (%)	Progressive disease *n* (%)
cRT_20–49 Gy_	69	0.0	18 (26.1)	38 (55.1)	13 (18.8)
cRT_50–60 Gy_	20	4 (20.0)	9 (45.0)	6 (30.0)	1 (5.0)
SBRT	36	2 (5.6)	22 (61.1)	12(33.3)	0.0
Brachytherapy	7	0.0	3 (42.9)	4 (57.1)	0.0
EQD2 < 40 Gy	48	0.0	12 (24.5)	30 (61.2)	7 (14.3)
EQD2 40–50 Gy	36	1 (2.9)	12 (34.3)	15 (42.9)	7(20.0)
EQD2 > 51 Gy	48	5 (10.4)	28 (58.3)	15 (31.3)	0.0
BED10 < 50 Gy	51	0.0	13 (25.5)	30 (58.8)	8 (15.7)
BED10 50–60 Gy	39	3 (7.7)	14 (35.9)	16 (41.0)	6 (15.4)
BED10 > 61 Gy	42	3 (7.1)	25 (59.5)	14 (33.3)	0.0

Second, we compared treatment efficacy depending on the location of the treated tumoural lesions. In all soft tissue lesions, objective response rate was about 50% (Supplementary Table 1). However, tTTP varied slightly between the different locations (median tTTP for local recurrence 9.8 months, 17.5 months for bone lesions, 17.2 months for pulmonary lesions and 13.5 months for liver lesions, *p* = 0.81).

In univariable analysis the following factors were associated with improved outcome: modality of RT, male sex, Ki67 index of the primary tumour ≤15%, absence of autonomous cortisol secretion, size of the treated lesion ≤30 mm, and, a time interval between primary diagnosis and RT > 12 months (Table 4). Using a multivariable model (with the ‘cRT _20-49_ Gy’ group as reference), time to local progression was significantly longer for cRT_50-60Gy_ (HR 0.01; 95% CI 0.03–0.33; *p* < 0.001) and for SBRT (HR 0.31; 95% CI 0.12–0.80; *p* = 0.016), but not for BT (HR 0.66; 95% CI 0.22–1.99; *p* = 0.46). Among the other variables, only glucocorticoid excess remained significant (Table 4).

**Table 4:** Predictive factors for tTTP in 132 lesions.

		Median tTTP (months)	Univariable analysis			Multivariable analysis		
	n		HR	95% CI	*P*	HR	95% CI	*P*
Treatment group								
1 cRT _20–49 Gy_	69	7.9	1			1		
2 cRT _50–60Gy_	20	Not reached	0.20	0.078–0.53	0.001	0.01	0.03–0.33	**<0.001**
3 SBRT	36	19.3	0.43	0.23–0.81	0.009	0.31	0.12–0.80	**0.016**
4 BT	7	4.6	0.97	0.35–2.73	0.96	0.66	0.22–1.99	0.46
Median age at start RT								
≤51	69	15.9	1					
>51	63	17.5	0.81	0.48-1.33	0.39			
Sex								
Female	74	15.1	1			1		
Male	58	28.6	0.63	0.37–1.1	0.07	0.48	0.22–1.03	0.62
KI67								
>15%	52	7.6	1			1		
≤15%	72	41.8	0.39	0.23–0.67	0.001	0.82	0.42–1.60	0.56
glucocorticoid excess								
yes	29	7.8	1			1		
no	103	19.2	0.47	0.27–0.81	0.006	0.48	0.24–0.99	**0.046**
Localisation								
1 LR	22	9.8	1					
2 pulmonary	32	17.5	1.1	0.51–2.31	0.82			
3 liver	12	17.2	0.97	0.42–2.27	0.96			
4 bone	46	13.5	1.5	0.59–3.58	0.42			
size of the treated lesion								
>30 mm	44	7.9	1			1		
≤30 mm	54	19.3	0.52	0.29–0.92	0.026	0.79	0.35–1.81	0.58
time primary diagnosis - RT								
≤12 months	24	6.7	1			1		
>12 months	108	18.1	0.53	0.29–0.97	0.04	0.61	0.26–1.42	0.25
number of therapies before RT								
≤3	42	9.7	1					
>3	90	16.5	0.85	0.50–1.45	0.56			
mitotane plasma level during RT								
≤14 mg/l	38	14.7	1					
>14 mg/l	91	18.1	0.92	0.52–1.62	0.77			

Only factors that showed at least a trend in the univariate analysis with *p* < 0.1 were further investigated by multivariate analysis. HR, Hazard ratio, LR local recurrence.

Median overall PFS in the cRT_50-60Gy_ group was 15.7 months, in the cRT_20-49 Gy_ group 5.6 months, in the SBRT group 3.2 months and in the BT group 2.9 months (*p* = 0.058). However, when adjusted in a multivariable analysis (with the ‘cRT_20-49_ Gy’ group as reference) these differences were not longer significant (cRT_50-60Gy_ HR 0.50; 95% CI 0.25–1.01; *p* = 0.054); SBRT HR 1.21; 95% CI 0.73–2.0; *p* = 0.47; and BT (HR 1.60; 95% CI 0.66–3.91; *p* = 0.29) (Supplementary Table 2). Again only the presence of glucocorticoid excess correlated with a significantly shorter oPFS (*p* = 0.012).

At last follow-up, 8 (40%) patients in the cRT_50-60Gy_ group, 9 (13%) patients in the cRT_20-49Gy_ group, 9 (25%) patients in the SBRT group and 3 (42%) patients in the BT group were still alive. Median overall survival in the cRT_50-60Gy_ group was 67.5 months, for cRT_20-49Gy_ 13.5 months, in the SBRT group 60.7 months and in the BT group 16.3 months (*p* < 0.001). After multivariate analysis overall survival in comparison to cRT_20-49Gy_ was significantly longer for cRT_50-60Gy_ (HR 0.36; 95% CI 0.16–0.83; *p* = 0.017), but not for SBRT (HR 0.45; 95% CI 0.18–1.12; *p* = 0.09) and BT group (HR 0.92; 95% CI 0.26–3.28; *p* = 0.91). Furthermore, Ki67 index >15% led to a significantly shorter OS (*p* = 0.014) (Supplementary Table 3).

### Clinical outcomes according to EQD2 and BED10

We analysed clinical outcomes in another approach distributing the lesions according to EQD2 and BED10. EQD2 was calculated for each RT treatment and the lesions were distributed in three groups EQD2_<40Gy_ (*n* = 48), EQD2_40-50Gy_ (*n* = 36), EQD2_>51Gy_ (*n* = 48). For BED10 lesions were distributed the cases in the following three groups <50 Gy (*n* = 51), 50–60 Gy (*n* = 39), >61 Gy (*n* = 42).

With regard to best objective response, EQD2 seems to represent a quite useful predictor, because 5 of 6 patients with complete response were treated with an EQD2 > 51 Gy. In addition, none of the 48 patients in this group experienced an immediate progress of the treated lesion. This was also reinforced by the multivariable adjusted Cox regression model (Supplementary Table 4 and Supplementary Fig. 1).

Using EQD2_<40Gy_ as reference group, time to local progression was significantly longer in the EQD2_>51Gy_ and EQD2_40-50Gy_ group (62.6 months vs. 19.2 months vs. 7.9 months; *p* < 0.001). After multivariable adjustment the corresponding HR were 0.24; 95% CI 0.09–0.64; *p* = 0.004 and 0.33; 95% CI 0.12–0.89; *p* = 0.029. Surprisingly male sex correlated with a significantly longer tTTP (HR 0.46; 95% CI 0.22–0.98; *p* = 0.045).

Regarding overall survival, patients with higher EQD2 had a significant longer OS in comparison to EQD2_<40 Gy_ (EQD2_50-60Gy_ HR 0.39; 95% CI 0.17–0.88; *p* = 0.023; EQD2_>61Gy_ HR 0.35; 95% CI 0.15–0.81; *p* = 0.014; multivariate analysis with the same variables as for overall survival in treatment groups). Among the other evaluated variables, only Ki67index > 15 % correlated with a shorter OS (*p* = 0.015).

Similar results were achieved if we dived the group using BED10. In comparison with BED10_<50Gy_, tTTP was significantly longer for BED10_50-60Gy_ (multivariable adjusted HR 0.25; 95% CI 0.09–0.68; *p* = 0.007) and for BED10_>61Gy_ (HR 0.29; 95% CI 0.12–0.77; *p* = 0.012) corresponding with a median tTTP of 28.6, 19.3, and 7.9 months (*p* = 0.001). Again, male sex was associated significantly with longer tTTP (*p* = 0.046). For overall survival, patients with higher BED had a significantly prolonged OS in comparison to BED_<50Gy_ (BED10_50-60Gy_ HR 0.41; 95% CI 0.18–0.93; *p* = 0.033; BED10_>61Gy_ HR 0.34; 95% CI 0.16–0.77; *p* = 0.009) (Supplementary Table 5 and Supplementary Fig. 1).

### Clinical outcomes according to the potential intention of treatment

We defined definitive intention corresponding treatment group cRT_50-60Gy_, SBRT and BT (*n* = 63) and treatment with cRT_20-49Gy_ (*n* = 69) as palliative treatment.

In the 63 lesions treated with ‘potentially definitive approach’, only 22 progressed during follow-up (34.9%), whereas this was the case in 40 of 69 lesions in ‘potentially palliative approach’ (58.0%). Accordingly, median tTTP was significantly different in these groups (62.3 months vs. 7.9 months; *p* < 0.001) and this difference was confirmed in the multivariable Cox regression model (HR 0.24; 95% CI 0.11–0.52; *p* < 0.001). We did not find any predictive factor among the other variables tested (Supplementary Fig. 2).

Overall survival was also significantly longer in the definitive treatment group (HR 0.44; 95% CI 0.22–0.89; *p* = 0.022). Similarly to the analysis for OS above, Ki67 index >15% led to a shorter OS (HR 0.41; 95% CI 0.20–0.84; *p* = 0.015) (Supplementary Fig. 2).

### Adverse events in patients with radiotherapy

The documented adverse events associated with RT were mostly mild or moderate and typical for RT (Table 5). The most frequent toxicities were low grade intestinal and pulmonary adverse events. One grade 3 intestinal adverse event with diarrhoea occurred after a conventional RT with 55 Gy, and two pulmonary grade 3 events with pneumonitis were documented after the conventional treatment with 43 Gy each, respectively.

**Table 5:** Adverse events according to the RTOG and CTCAE criteria (33).

Adverse event	Grade 1	Grade 2	Grade 3
Intestinal	13	5	1
Renal	5		
Hepatic	2		
Pulmonary	16	4	2
Skin	2	1	
Musculoskeletal		1	
Anaemia	2		
Fatigue	3	2	

## Discussion

This retrospective study represents the largest cohort of patients with advanced ACC treated with RT. Our data indicate that RT (especially when applied in adequate dosage) is of benefit for selected patients confirming what previous small studies [19, 23–25, 32] suggested and what the recent guideline panels [1, 8] concluded mainly based on expert opinion. In most of the treated lesions stable disease or partial responses could be documented; in 6 out of 132 lesions even complete response was achieved. Overall, in only 11% of lesions immediate progression was diagnosed. The reported toxicity was moderate and within the expected range of RT of solid tumoural lesions.

The comparison of the different RT modalities suggests that conventional RT with more than 50 Gy is more efficient than the other methods, as expected. However, the small number of patients in this group (*n* = 20) and especially in the brachytherapy group (*n* = 7) calls for caution. To allow a better comparability of the different fractionation schemes we applied the concept of the linear quadratic model and calculated the corresponding EQD2 and BED10 values. These two approaches reinforced our findings of longer tTTP with higher radiation dosage. Of note, patients treated with an EQD2 > 40 Gy or BED10 > 50 Gy experienced a median time to local progression of 62.6 and 28.6 months respectively. Thus, one could also conclude that SBRT might be the preferred option if feasible and the achievable EQD2 and BED10 are above these cutoffs. Furthermore, patient preference is likely in favour of SBRT because of the shorter overall treatment period.

RT in ACC is traditionally used as a palliative therapy especially in symptomatic bone, brain or inferior vena cava involvement [35]. However, our data clearly indicate its important role in selected patients with ACC and non-resectable lesions beyond palliation. Nevertheless, a relevant proportion of patients of our cohort has been treated with a palliative concept not primarily aiming at long-term disease control. Therefore, it appears to be important to adjust for possible prognostic or predictive factors. However, even with this approach, conventional RT with more than 50 Gy seemed to be the most efficient therapy. Unfortunately, our search for predictive factors was not very successful. Overall, there was some indication that patients without glucocorticoid excess and with a Ki67 ≤ 15% of the primary tumour might benefit more than others. However, we would be reluctant to use these factors as strong selection criteria. Similarly, we are not yet convinced that male patients have a better outcome to RT, although this finding might deserve future research.

Although RT cannot prevent progression of other lesions or further metastatic spread, it has clearly the power to reach long-term disease control in many patients with limited numbers of tumoural lesions. Therefore, it seems likely that this local effect also translates to an overall clinical benefit in a relevant subset of patients, although we cannot prove this in this retrospective analysis. Furthermore, RT could have additional positive effects in patients treated with immunotherapy due to potential abscopal effects. However, this has never been demonstrated for patients with ACC.

Our study has obvious limitations including the retrospective nature, the still low number of patients, and the lack of a control group. However, due to the absence of published evidence on RT in advanced ACC, this study currently represents the best data available. Due to the rarity of the disease, it is unlikely that a much larger cohort will be recruited in the near future. Another limitation is the heterogeneity of RT modalities and the different group sizes. In addition, we have to acknowledge that the decision for RT was made by local treating physicians and was not based on any defined criteria. Therefore, a selection bias is possible. Smaller lesions and lesions with lower Ki67 index were treated in most of the cases with higher doses of RT, which might have affect the response to RT. Furthermore, RT was not applied according to a standardised protocol nor was this the case for co-treatment with mitotane. However, the number of patients treated with mitotane was quite similar between the four groups and the documented mitotane plasma levels were in the same range. Neither the treatment with mitotane nor the mitotane plasma level had any relevant influence on the efficacy of RT.

In conclusion, our study clearly suggests that RT is associated with beneficial effects on clinical outcome in selected patients with advanced ACC when applied in adequate dosage. These results provide the strongest evidence so far for the use of RT in advanced ACC and it provides a good basis for prospective studies to reduce the uncertainties and limitations of retrospective cohort studies. We would hope that our study will raise interest in considering RT more frequently as a treatment option in advanced disease, where it should not be limited to palliation.

## Supplementary information

Supplementary figure 1

Supplementary figure 2

Supplementary Table 1

Supplementary Table 2

Supplementary Table 3

Supplementary Table 4

Supplementary table 5

## Data Availability

The datasets generated and /or analysed during this study are not publicly available due to privacy issues of the patients with a very rare disease but are available in an anonymized fashion from the corresponding author on reasonable request.
