# Supplementary figures and images for: Efficacy and safety of radiation therapy in advanced adrenocortical carcinoma
Source: Br J Cancer. 2022 Dec 8;128(4):586–93. doi: 10.1038/s41416-022-02082-0 (PMC9938283; doi:10.1038/s41416-022-02082-0)

## Slide 1
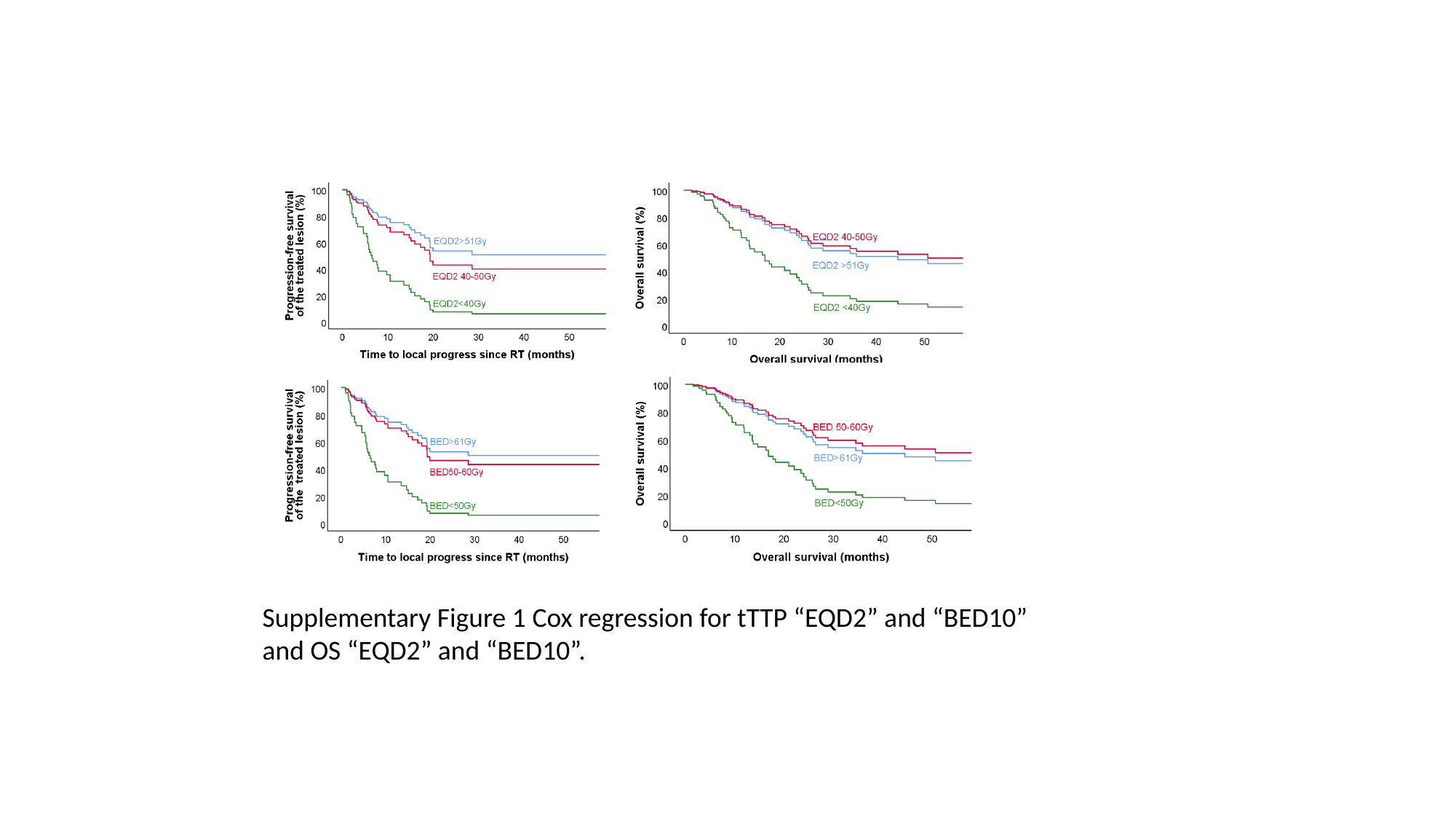

Supplementary Figure 1 Cox regression for tTTP “EQD2” and “BED10” and OS “EQD2” and “BED10”.

Supplement: Supplementary file 1 — Supplementary figure 1 [file 41416_2022_2082_MOESM1_ESM.pptx]
