# Supplementary material for: Efficacy and safety of radiation therapy in advanced adrenocortical carcinoma
Source: Br J Cancer. 2022 Dec 8;128(4):586–93. doi: 10.1038/s41416-022-02082-0 (PMC9938283; doi:10.1038/s41416-022-02082-0)
Supplement: Supplementary file 2 — Supplementary figure 2 [file 41416_2022_2082_MOESM2_ESM.pptx]

## Slide 1
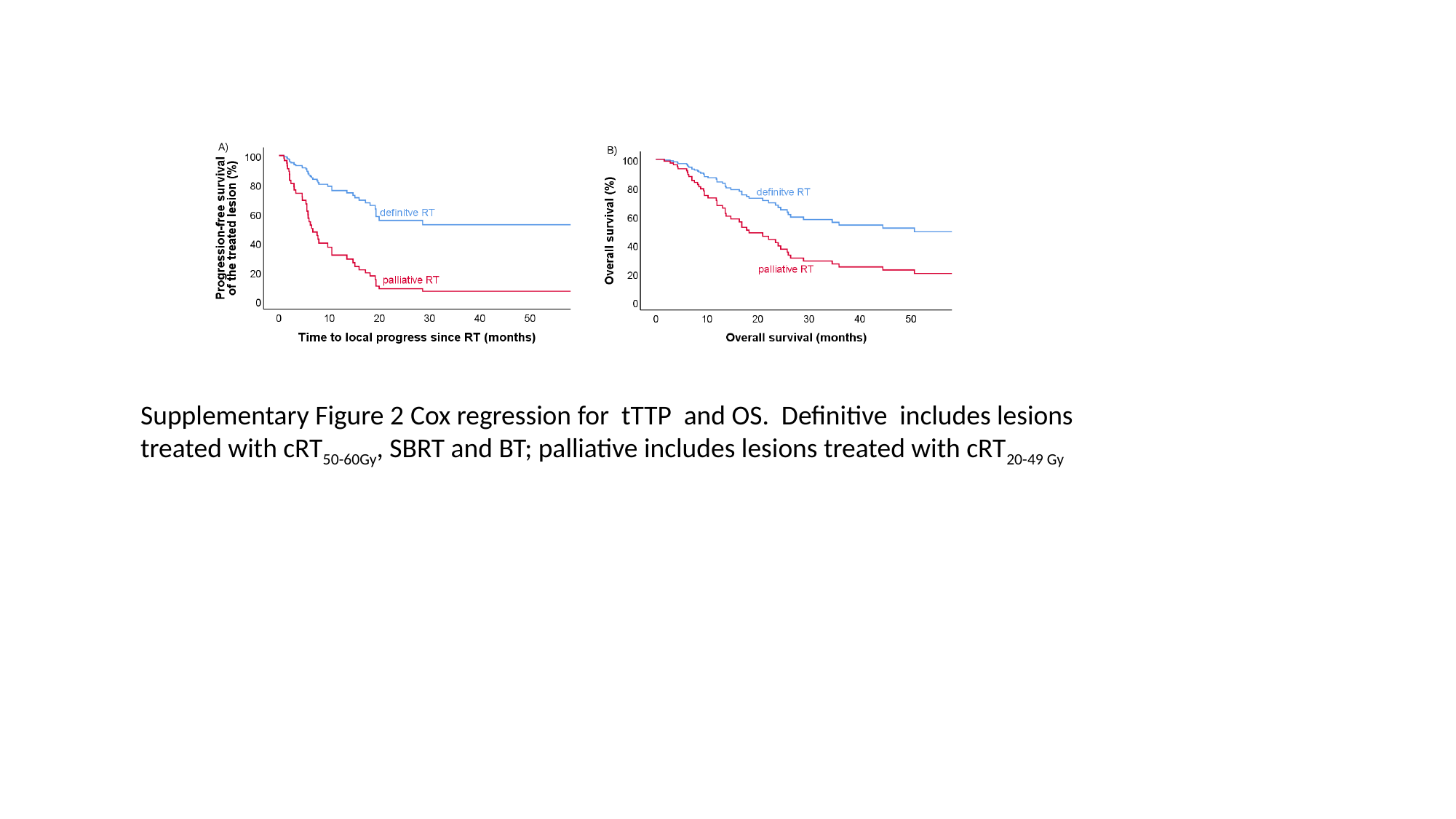

Supplementary Figure 2 Cox regression for tTTP and OS. Definitive includes lesions treated with cRT50-60Gy, SBRT and BT; palliative includes lesions treated with cRT20-49 Gy
