# Supplementary material for: Efficacy and safety of radiation therapy in advanced adrenocortical carcinoma
Source: Br J Cancer. 2022 Dec 8;128(4):586–93. doi: 10.1038/s41416-022-02082-0 (PMC9938283; doi:10.1038/s41416-022-02082-0)
Supplement: Supplementary file 3 — Supplementary Table 1 [file 41416_2022_2082_MOESM3_ESM.docx]

**Supplementary Table 1 Objective response rates per treated lesion.**

| Localisation of treated lesion | Complete response n (%) | Partial response n (%) | Stable disease n (%) | Progressive disease n (%) |
| --- | --- | --- | --- | --- |
| Local recurrence, n=22 | 3 (13.6) | 8 (36.4) | 9 (40.9) | 2 (9.1) |
| Lung, n=32 | 1 (3.1) | 17 (53.1) | 12 (37.5) | 2 (6.3) |
| Liver, n=16 | 1 (6.3) | 7 (43.8) | 6 (37.5) | 2 (12.5) |
| Bone, n=46 | 0 | 11 (23.9) | 29 (63) | 6 (13) |
| Lymph node, n=3 | 0 | 2 (66.7) | 1 (33.3) | 0 |
| Brain, n=4 | 1 (25) | 3 (75) | 0 | 0 |
| Other soft tissue, n=9 | 0 | 4 (44.4) | 3 (33.3) | 2 (22.3) |
