# Supplementary material for: Efficacy and safety of radiation therapy in advanced adrenocortical carcinoma
Source: Br J Cancer. 2022 Dec 8;128(4):586–93. doi: 10.1038/s41416-022-02082-0 (PMC9938283; doi:10.1038/s41416-022-02082-0)
Supplement: Supplementary file 4 — Supplementary Table 2 [file 41416_2022_2082_MOESM4_ESM.docx]

**Supplementary Table 2 Predictive factors for oPFS, in total 132 lesions.**

|  |  | **Median oPFS** | **Univariable Analysis** | | | **Mutlivariable Analysis** | | |
| --- | --- | --- | --- | --- | --- | --- | --- | --- |
|  | n | **(months)** | HR | 95% CI | ***P*** | HR | 95% CI | ***P*** |
| Treatment group  1 cRT _20-49 Gy_  2 cRT _50-60Gy_  3 SBRT  4 BT | 69  20  36  7 | 5.6  15.7  3.2  2.9 | 1  0.45  1.04  1.24 | 0.23-0.86  0.66-1.65  0.53-2.91 | 0.015  0.86  0.62 | 1  0.50  1.21  1.60 | 0.25-1.01  0.73-2.0  0.66-3.91 | 0.054  0.47  0.29 |
| Median age at start RT  ≤ 51  > 51 | 69  63 | 5.2  5.5 | 1  0.81 | 0.54-1.20 | 0.28 |  |  |  |
| Sex  female  male | 74  58 | 5.1  6.6 | 1  0.89 | 0.59-1.32 | 0.55 |  |  |  |
| KI67  >15%  ≤15% | 52  72 | 4.9  6.9 | 1  0.58 | 0.38-0.89 | 0.012 | 1  0.66 | 0.41-1.04 | 0.08 |
| glucocorticoid excess  yes  no | 29  103 | 3.6  7.5 | 1  0.46 | 0.29-0.74 | 0.001 | 1  0.52 | 0.31-0.86 | **0.012** |
| Localisation  1 LR  2 pulmonary  3 liver  4 bone | 22  32  12  46 | 4.9  7.5  4.1  7.9 | 1  0.87  0.86  1.2 | 0.41-1.84  0.45-1.67  0.58-2.38 | 0.72  0.66  0.65 |  |  |  |
| size of treated lesion  >30 mm  ≤30 mm | 44  54 | 3.8  5.7 | 1  0.85 | 0.53-1.34 | 0.47 |  |  |  |
| Number of metastases  >5  ≤5 | 71  60 | 7.9  3.8 | 1  0.62 | 0.42-0.93 | 0.02 | 1  0.65 | 0.43-1.01 | 0.054 |
| time primary diagnosis - RT  ≤ 12 months  > 12 months | 24  108 | 3.0  6.4 | 1  1.4 | 0.83-2.3 | 0.22 |  |  |  |
| number of therapies before RT  ≤3  >3 | 42  90 | 6.2  4.9 | 1  0.71 | 0.46-1.1 | 0.11 |  |  |  |
| mitotane plasma level during RT  ≤14 mg/l  >14 mg/l | 38  91 | 6.4  4.9 | 1  0.96 | 0.63-1.48 | 0.86 |  |  |  |

Only factors that showed at least a trend in the univariable analysis with p<0.1 were further investigated by multivariable analysis. HR, Hazard ratio; LR local recurrence, RT radiotherapy.
