# Supplementary material for: Efficacy and safety of radiation therapy in advanced adrenocortical carcinoma
Source: Br J Cancer. 2022 Dec 8;128(4):586–93. doi: 10.1038/s41416-022-02082-0 (PMC9938283; doi:10.1038/s41416-022-02082-0)
Supplement: Supplementary file 5 — Supplementary Table 3 [file 41416_2022_2082_MOESM5_ESM.docx]

**Supplementary Table 3 Predictive factors for OS, in total 132 lesions**

|  |  | Median OS (months) | Univariable analysis |  |  | Mutlivariable analysis |  |  |
| --- | --- | --- | --- | --- | --- | --- | --- | --- |
|  | n |  | HR | 95% CI | ***P*** | HR | 95% CI | ***P*** |
| Treatment group  1 cRT _20-49 Gy_  2 cRT _50-60Gy_  3 SBRT  4 BT | 69  20  36  7 | 13.5  67.5  60.7  16.3 | 1  0.35  0.32  0.73 | 0.18-0.67  0.18-0.57  0.23-2.35 | 0.002  0.001  0.59 | 1  0.36  0.45  0.92 | 0.16-0.83  0.18-1.12  0.26-3.28 | **0.017**  **0.09**  0.91 |
| Median age at start RT  ≤ 51  > 51 | 69  63 | 24.0  41.8 | 1  1.40 | 0.91-2.2 | 0.13 |  |  |  |
| Sex  female  male | 74  58 | 22.1  41.3 | 1  0.63 | 0.41-0.98 | 0.042 | 1  1.33 | 0.66-2.86 | 0.43 |
| KI67  >15%  ≤15% | 52  72 | 15.1  52.1 | 1  0.28 | 0.17-0.46 | <0.001 | 1  0.39 | 0.19-0.83 | **0.014** |
| glucocorticoid excess  yes  no | 29  103 | 18.3  39.4 | 1  0.45 | 0.27-0.76 | 0.003 | 1  0.55 | 0.27-1.14 | 0.11 |
| Localisation  1 LR  2 pulmonary  3 liver  4 bone | 22  32  12  46 | 25.9  20.9  50.7  13.6 | 1  0.84  1.19  0.50 | 0.36-2.16  0.53-2.67  0.19-1.27 | 0.79  0.68  0.15 |  |  |  |
| size of treated lesion  >30 mm  ≤30 mm | 44  54 | 44.5  13.7 | 1  0.49 | 0.29-0.84 | 0.009 | 1  0.67 | 0.34-1.31 | 0.24 |
| Number of metastases  >5  ≤5 | 71  60 | 24.0  28.9 | 1  1.10 | 0.68-1.62 | 0.83 |  |  |  |
| time primary diagnosis - RT  ≤ 12 months  > 12 months | 24  108 | 13.8  35.9 | 1  2.03 | 1.22-3.37 | 0.006 | 1  1.49 | 0.74-3.04 | 0.26 |
| number of therapies before RT  ≤3  >3 | 42  90 | 18.4  35.9 | 1  1.26 | 0.81-1.97 | 0.31 |  |  |  |
| mitotane plasma level during RT  ≤14 mg/l  >14 mg/l | 38  91 | 34.6  25.7 | 1  1.10 | 0.62-1.62 | 0.99 |  |  |  |

Only factors that showed at least a trend in the univariable analysis with p<0.1 were further investigated by multivariable analysis. HR, Hazard ratio; LR local recurrence, RT radiotherapy.
