# Supplementary material for: Efficacy and safety of radiation therapy in advanced adrenocortical carcinoma
Source: Br J Cancer. 2022 Dec 8;128(4):586–93. doi: 10.1038/s41416-022-02082-0 (PMC9938283; doi:10.1038/s41416-022-02082-0)
Supplement: Supplementary file 6 — Supplementary Table 4 [file 41416_2022_2082_MOESM6_ESM.docx]

**Supplementary Table 4 Predictive factors for tTTP per lesion EQD2, in total 132 lesions.**

|  |  | Median tTTP (months) | Univariate Analysis |  |  | Mutlivariate Analysis |  |  |
| --- | --- | --- | --- | --- | --- | --- | --- | --- |
|  | n |  | HR | 95% CI | ***P*** | HR | 95% CI | ***P*** |
| EQD2  <40Gy  40-50Gy  >51Gy | 48  36  48 | 7.9  19.2  62.6 | 1  0.32  0.55 | 0.17-0.59  0.29-1.1 | <0.001  0.054 | 1  0.24  0.33 | 0.09-0.64  0.12-0.89 | 0.004  0.029 |
| Median age at start RT  ≤ 51  > 51 | 69  63 | 15.9  17.5 | 1  0.81 | 0.48-1.33 | 0.39 |  |  |  |
| Sex  female  male | 74  58 | 15.1  28.6 | 1  0.63 | 0.37-1.1 | 0.07 | 1  0.46 | 0.22-0.98 | **0.045** |
| KI67  >15%  ≤15% | 52  72 | 7.6  41.8 | 1  0.39 | 0.23-0.67 | 0.001 | 1  0.87 | 0.44-1.74 | 0.70 |
| glucocorticoid excess  yes  no | 29  103 | 7.8  19.2 | 1  0.47 | 0.27-0.81 | 0.006 | 1  0.65 | 0.32-1.29 | 0.22 |
| Localisation  1 LR  2 pulmonary  3 liver  4 bone | 22  32  12  46 | 9.8  17.5  17.2  13.5 | 1  1.1  0.97  1.5 | 0.51-2.31  0.42-2.27  0.59-3.58 | 0.82  0.96  0.42 |  |  |  |
| size of treated lesions  >30 mm  ≤30 mm | 44  54 | 7.9  19.3 | 1  0.52 | 0.29-0.92 | 0.026 | 1  0.85 | 0.39-1.84 | 0.67 |
| time primary diagnosis - RT  ≤ 12 months  > 12 months | 24  108 | 6.7  18.1 | 1  0.53 | 0.29-0.97 | 0.04 | 1  0.88 | 0.34-2.29 | 0.79 |
| number of therapies before RT  ≤3  >3 | 42  90 | 9.7  16.5 | 1  0.85 | 0.50-1.45 | 0.56 |  |  |  |
| mitotane plasma level during RT  ≤14 mg/l  >14 mg/l | 38  91 | 14.7  18.1 | 1  0.92 | 0.52-1.62 | 0.77 |  |  |  |

Only factors that showed at least a trend in the univariable analysis with p<0.1 were further investigated by multivariable analysis. HR, Hazard ratio; LR local recurrence, RT radiotherapy.
