# Supplementary material for: Efficacy and safety of radiation therapy in advanced adrenocortical carcinoma
Source: Br J Cancer. 2022 Dec 8;128(4):586–93. doi: 10.1038/s41416-022-02082-0 (PMC9938283; doi:10.1038/s41416-022-02082-0)
Supplement: Supplementary file 7 — Supplementary table 5 [file 41416_2022_2082_MOESM7_ESM.docx]

**Supplementary Table 5 Predictive factors for tTTP per lesion BED10, in total 132 lesions.**

|  |  | Median tTTP (months) | Univariate analysis |  |  | Mutlivariate analysis |  |  |
| --- | --- | --- | --- | --- | --- | --- | --- | --- |
|  | n |  | HR | 95% CI | ***P*** | HR | 95% CI | ***P*** |
| BED10  <50Gy  50-60Gy  >61Gy | 51  39  42 | 7.9  28.6  19.3 | 1  0.35  0.47 | 0.18-0.66  0.25-0.86 | 0.001  0.014 | 1  0.25  0.29 | 0.090-0.68  0.12-0.77 | **0.007**  **0.012** |
| Median age at start RT  ≤ 51  > 51 | 69  63 | 15.9  17.5 | 1  0.81 | 0.48-1.33 | 0.39 |  |  |  |
| Sex  female  male | 74  58 | 15.1  28.6 | 1  0.63 | 0.37-1.1 | 0.07 | 1  0.47 | 0.22-0.97 | **0.046** |
| KI67  >15%  ≤15% | 52  72 | 7.6  41.8 | 1  0.39 | 0.23-0.67 | 0.001 | 1  0.86 | 0.43-1.71 | 0.67 |
| glucocorticoid excess  yes  no | 29  103 | 7.8  19.2 | 1  0.47 | 0.27-0.81 | 0.006 | 1  0.64 | 0.32-1.29 | 0.21 |
| Localisation  1 LR  2 pulmonary  3 liver  4 bone | 22  32  12  46 | 9.8  17.5  17.2  13.5 | 1  1.1  0.97  1.5 | 0.51-2.31  0.42-2.27  0.59-3.58 | 0.82  0.96  0.42 |  |  |  |
| size treated lesion  >30 mm  ≤30 mm | 44  54 | 7.9  19.3 | 1  0.52 | 0.29-0.92 | 0.026 | 1  0.85 | 0.39-1.89 | 0.69 |
| time primary diagnosis - RT  ≤ 12 months  > 12 months | 24  108 | 6.7  18.1 | 1  0.53 | 0.29-0.97 | 0.04 | 1  0.82 | 0.33-2.10 | 0.67 |
| number of therapies before RT  ≤3  >3 | 42  90 | 9.7  16.5 | 1  0.85 | 0.50-1.45 | 0.56 |  |  |  |
| mitotane plasma level during RT  ≤14 mg/l  >14 mg/l | 38  91 | 14.7  18.1 | 1  0.92 | 0.52-1.62 | 0.77 |  |  |  |

Only factors that showed at least a trend in the univariable analysis with p<0.1 were further investigated by multivariable analysis. HR, Hazard ratio; LR local recurrence, RT radiotherapy.
